# Optimizing COVID-19 vaccine distribution across the United States using deterministic and stochastic recurrent neural networks

**DOI:** 10.1371/journal.pone.0253925

**Published:** 2021-07-06

**Authors:** Mohammad Reza Davahli, Waldemar Karwowski, Krzysztof Fiok

**Affiliations:** Department of Industrial Engineering and Management Systems, University of Central Florida, Orlando, Florida, United States of America; Universita degli Studi di Pisa, ITALY

## Abstract

Optimizing COVID-19 vaccine distribution can help plan around the limited production and distribution of vaccination, particularly in early stages. One of the main criteria for equitable vaccine distribution is predicting the geographic distribution of active virus at the time of vaccination. This research developed sequence-learning models to predict the behavior of the COVID-19 pandemic across the US, based on previously reported information. For this objective, we used two time-series datasets of confirmed COVID-19 cases and COVID-19 effective reproduction numbers from January 22, 2020 to November 26, 2020 for all states in the US. The datasets have 310 time-steps (days) and 50 features (US states). To avoid training the models for all states, we categorized US states on the basis of their similarity to previously reported COVID-19 behavior. For this purpose, we used an unsupervised self-organizing map to categorize all states of the US into four groups on the basis of the similarity of their effective reproduction numbers. After selecting a leading state (the state with earliest outbreaks) in each group, we developed deterministic and stochastic Long Short Term Memory (LSTM) and Mixture Density Network (MDN) models. We trained the models with data from each leading state to make predictions, then compared the models with a baseline linear regression model. We also remove seasonality and trends from a dataset of non-stationary COVID-19 cases to determine the effects on prediction. We showed that the deterministic LSTM model trained on the COVID-19 effective reproduction numbers outperforms other prediction methods.

## Introduction

The supply of approved vaccines for the COVID-19 will be limited in early stages [1]. Therefore, the basic question of what the optimized vaccine distribution might be must be answered. Regarding this question, the Centers for Disease Control and Prevention (CDC) and the National Institutes of Health (NIH) asked the National Academies to perform a consensus study on the equitable allocation of the COVID-19 vaccines among potential recipients [2]. The study takes different factors into account, such as high-risk individuals, population health disparities, assuring communities of color about the vaccine, addressing vaccine hesitancy among individuals, and considering ethical values [3, 4]. However, one of the main criteria for optimizing vaccine distribution is the geographic spread of the active virus at the time of vaccination [2]. Therefore, predicting the future spreading patterns of virus across different regions is important.

In this article, we develop real-time approaches for predicting the behavior of COVID-19 in all US states. We use data from the Centers for Disease and Prevention website and create two time-series datasets of the number of confirmed cases, and the effective reproduction numbers for all US states. The effective reproduction number, R_t_, is defined as “the average number of secondary cases of disease caused by a single infected individual over her or his infectious period” [5].

To avoid training the models for all states, we use a self-organizing map (SOM) [6] to categorize all states into four groups according to their similarity in the reported effective reproduction numbers. In each group, we select the leading state (the state with earliest outbreaks). A deterministic Long Short Term Memory (LSTM) model [7], recurrent neural network (RNN) model, and stochastic Mixture Density Network (MDN) model [8] are then trained on data from each of the leading states.

In the deterministic LSTM model, the network output is the number of confirmed cases and the value of effective reproduction number in the next time-step. We use an LSTM RNN because (1) more confirmed cases can lead to more potential infection among populations in the future, and therefore, retaining all relevant historical information is important, and (2) this intelligent sequence analysis model has been reported by several studies to have high efficiency in time series forecasting problems [9].

In the stochastic MDN model, the network output is parameters of mixture distributions rather than a direct prediction value. The proposed MDN model is a combination of LSTM layers and a mixture of distributions. In this model, LSTM layers supply parameters for one or several distributions, which are then combined with weighting [8]. Finally, a sample of data can be extracted from the developed mixture distributions as an actual prediction [10].

We then compare the performance of developed models with a baseline linear regression model [11]. We aim to study whether using deterministic and stochastic sequence-learning models might have better predictive performance than linear regression. We also use an Augmented Dickey Fuller test [12] to assess the stationary and non-stationary status of the input dataset. We then remove seasonality and trend from the non-stationary datasets to investigate their effects on predictive performance.

This article is structured as follows. Section two discusses a published article on using artificial intelligence and machine learning to predict the behavior of the COVID-19 pandemic. Section three presents a brief mathematical explanation of R_t_, seasonal-trend decomposition, SOMs, RNNs, and mixture density networks (MDNs). Section four discusses the development of sequence learning predictive models. Finally, section five explains the experimental setup, performance metrics, and results.

## Literature review

On December 8, 2019, the government of China reported treatment of several new virus cases of a disease later named coronavirus disease 2019 (COVID-19) [13]. Since then, COVID-19 has spread across many countries and become a pandemic. COVID-19 is a highly transmissible respiratory disease with symptoms such as cough, fever, and breathing problems; it spreads through contact with infected individuals [14]. In January 2020, the US reported its first confirmed case of COVID-19; in mid-February 2020, the COVID-19 pandemic began to cause unprecedented social and economic consequences [13]. On December 14, 2020, the CDC reported 16,113,148 confirmed COVID-19 cases and 298,266 deaths in the US [15]. In this dire situation, the successful prior application of artificial intelligence and machine learning in critical problems inspired researchers to use these techniques against the COVID-19 pandemic. Artificial intelligence and machine learning have been used in various areas of predicting, contact tracing, screening, forecasting, and drug development for the COVID-19 pandemic [16].

Ribeiro et al. [17] have used cumulative confirmed Brazilian COVID-19 cases to train a support vector regression algorithm to forecast case numbers 6 days in advance. Chakraborty and Ghosh [18] have developed a hybrid method based on a Wavelet-based forecasting model and autoregressive integrated moving average model to forecast case numbers 10 days in advance for France, India, Canada, South Korea, and the UK. Chakraborty and Ghosh [18] have indicated that these forecast numbers of COVID-19 cases can act as an early-warning for policymakers and can be useful for the efficient allocation of health care resources. Kapoor et al. [19] have used mobility data and Graph Neural Networks to predict COVID-19 cases and have reported a 6% lower root mean squared logarithmic error than the best-performing baseline models.

Hartono [20] has indicated that developing an efficient predictive model is difficult because of the unknown characteristics of the virus causing COVID-19, as well as the political and geographical influences. Hartono [20] has used a topological autoencoder (TA), a topological neural network, to map the transmission dynamics of COVID-19 spread in several countries. TA produces a two-dimensional map in which countries with similar transmission dynamics are located close to each other. After selection of a target location for forecasting, TA has been used to identify a reference location with similar transmission dynamics that experienced earlier spread of the virus causing COVID-19. Finally, LSTM has been trained on data from the reference location to forecast the COVID-19 distribution in the target location.

Tomar and Gupta [21] have used LSTM and curve fitting to predict the number of COVID-19 positive cases and the number of recovered cases in India 30 days in advance. In that study, the data were collected from January 30, 2020 to April 4, 2020; 80% of the data were used for training, and 20% were used for testing. Li et al. [22] have developed an integrated spatiotemporal model based on RNNs and epidemic differential equations to predict the number of COVID-19 cases in Italy 7 days in advance.

Arora et al. [9] have used RNN based LSTM variants including Deep LSTM, Bidirectional LSTM, and Convolutional LSTM to predict the number of COVID-19 cases in India 1 day and 1 week in advance. In that study, the states of India are categorized into different areas according to the daily growth rate and the number of confirmed COVID-19 cases. The dataset contains time-series data of confirmed COVID-19 cases from March 14, 2020 to May 14, 2020 for each state in India [9]. Arora et al. [9] have conducted an experiment on open source libraries and have used the Adam optimizer to optimize the mean squared error loss. The authors used the mean absolute percentage error (MAPE) to compare the performance of several predictive methods and found an average MAPE of 3.22% for bi-directional LSTM, 4.81% for Stacked LSTM, and 5.05% for conv-LSTM.

Shahid et al. [23] have used support vector regression, autoregressive integrated moving average, LSTM, and Bidirectional LSTM for predicting confirmed COVID-19 cases, deaths, and recoveries in Israel, Russia, Brazil, Spain, the UK, Germany, Italy, China, India, and the US. The study used the mean absolute error, root mean square error, and r^2^_score indices to measure the performance of the models. The methods were found to rank as follows from best performance to worst performance: Bidirectional LSTM, LSTM, support vector regression, and autoregressive integrated moving average.

Chimmula and Zhang [12] have collected data on the numbers of confirmed COVID-19 cases, of fatalities, and recovered patients in a time series format from the Canadian Health Authority and Johns Hopkins University. The Augmented Dickey Fuller test was used to identify the effects of trends on the dataset and to report the stationary and non-stationary nature of the data [12]. The study has also developed an LSTM model to forecast the pandemic outbreak in Canada.

## Mathematical models

In this section, the mathematical formulae of effective reproduction numbers, SOMs, RNNs, and MDNs are explained.

### Effective reproduction number

The effective reproduction number, R_t_, is defined as “the expected number of new infections caused by an infectious individual in a population where some individuals may no longer be susceptible” [24]. One of the main reasons for calculating R_t_ is to determine how interventions and control efforts in population immunity, policy, and other elements affect transmission in specific time-steps [25]. Furthermore, R_t_ can be used to study real-time changes in COVID-19 transmission [24]. To bring the pandemic under control, R_t_ must be decreased to less than 1 and as close to 0 as possible [5]. Therefore, predicting R_t_, which is situation- and time-specific, can aid in understanding the pathogen transmissibility during the COVID-19 pandemic in the future. Several methods have been developed to estimate R_t_ but we use the method of Cori et al. [5], in which the effective reproduction number is as follows:

$$R_{t}=\frac{I_{t}}{\sum_{s=1}^{t}I_{t-s}w_{s}}$$ (1)

where I_t_ is the number of incidents of infections on day t, and w_s_ is the generation interval, which is defined as “the time between the infection time of an infected person and the infection time of his or her infector” [26]. In this equation, the generation interval is the only parametric assumption adopted from Nishiura et al. [27]. That study obtained 28 infector-infectee pairs and used the log-normal distribution and the discretized gamma distributions to generate the results. Nishiura et al. [27] have reported the standard deviation and mean of the serial interval at 2.9 days (95% credible interval (CrI): 1.9, 4.9) and 4.7 days (95% CrI: 3.7, 6.0). For estimating R_t_, the Excel file of EpiEstim package was borrowed from Cori et al. [5] (Please refer to https://github.com/RezaDavahli for input data; 10 February 2021) [28].

### Seasonal-trend decomposition

Normally, time series data can be decomposed into the trend, seasonality, and residual, as represented in the following equation:

$$q=\tau_{t}+s_{t}+r_{t}$$ (2)

where t = 1, 2, · · ·, N; x_t_ is an original signal at time t; τ_t_ is the trend; s_t_ is the seasonality, which is the patterns that repeat with a period of time; and r_t_ is the residual. Several decomposition algorithms have been proposed for periodic and non-periodic datasets [29]. In this article, we use Seasonal-Trend Decomposition in six steps, which have been fully discussed by Qin et al. [30].

Before removing the seasonality and trend, we apply the Dickey Fuller test to determine whether the datasets are stationary or non-stationary. For the stationary dataset, seasonality and trend are not removed.

### Self-organizing map

Teuvo Kohonen developed the SOM as a new form of neural network architecture and learning algorithm in the 1980s [6]. SOM uses an unsupervised learning process to analyze and represent the basic structures of a dataset as a map [31]. Therefore, SOM is commonly used to convert high-dimensional datasets into one- or two-dimensional maps [32]. Suppose that the input variables are X = (x_1_,x_2_,⋯x_p_)′; the weight vector assigned to the node l is u_l_ = (u_l1_,u_l2_,⋯u_lp_) ′; u_lj_ is the weight associated with node l of input variable x_j_; and p is the number of input variables [33].

The learning concept of SOM involves detecting and moving the winning node closer to each training case. For this purpose, the Euclidean distance d_i_ between the weight vector and the input variables is calculated for each item i in the training case. Subsequently, the weights of the winning node with the smallest d_i_ are updated by a learning rule. In each step, the index q of the winning node is:

$$q=argmin\|u_{l}^{s}-x_{i}\|$$ (3)

where $u_{l}^{s}$ is the weight for the l_th_ node on the sth step, α^s^ is the learning rate for the sth step, and x_i_ is the input variable for the i_th_ training case. For the winner node, the update rule is:

$$u_{q}^{s+1}=u_{q}^{s}(1-\alpha^{s})+x_{i}\alpha^{s}=u_{q}^{s}+\alpha^{s}(x_{i}-u_{q}^{s})$$ (4)

where u_l_^s+1^ is set to u_l_^s^ for all non-winning nodes.

### Recurrent neural networks

**Fig 1:**
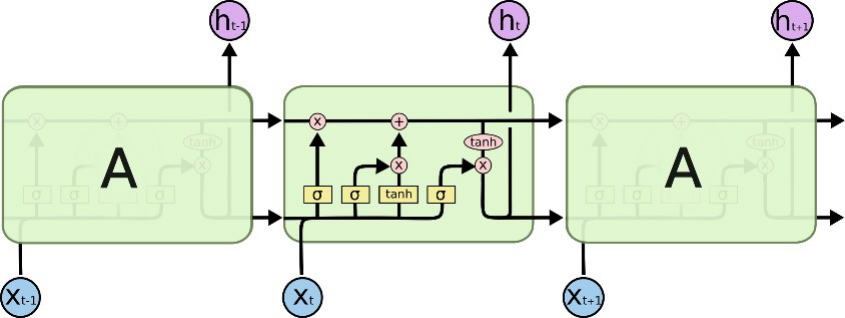
LSTM structure [36].

Deep learning methods are effective for prediction because they automatically extract appropriate features from datasets [34]. RNN, a deep learning method, can store extensive historical information and use it to accurately predict the next steps in time-series problems [35]. However, its main disadvantage is long training time, because of vanishing gradient problems [21]. To overcome this problem, the LSTM structure, comprising a cell, an input gate, an output gate, and a forget gate, was developed to consider a long-term dependency [7]. In this structure, the cell stores values over arbitrary time intervals, and the gates adjust the flow of information in the recurrent hidden layer, as represented in Fig 1 [21].

The states of an input gate, an output gate, and a forget gate can be demonstrated mathematically by five equations:

$$f_{t}=\sigma (W_{f}.[h_{t-1},x_{t}]+b_{t})$$ (5)

$$i_{t}=\sigma (W_{i}.[h_{t-1},x_{t}]+b_{i})$$ (6)

$${\tilde{C}}_{t}=tanh(W_{c}.[h_{t-1},x_{t}]+b_{c})$$ (7)

$$C_{t}=f_{t}*C_{t-1}+i_{t}*{\tilde{C}}_{t}$$ (8)

$$o_{t}=\sigma (W_{o}.[h_{t-1},x_{t}]+b_{o})$$ (9)

$$h_{t}=o_{t}*tanh(C_{t})$$ (10)

In these equations, σ is the logistic sigmoid activation function; C_t_ is the cell state; W indicates the weight matrices; and i, o, and f indicate the input gate, output gate, and forget gate, respectively [36]. In this structure, the input gate specifies the flow of information and protects the cell from irrelevant information, the forget gate deletes irrelevant information, and the output gate regulates the flow of information passing through the rest of the network [9].

### Mixture density networks

**Fig 2:**
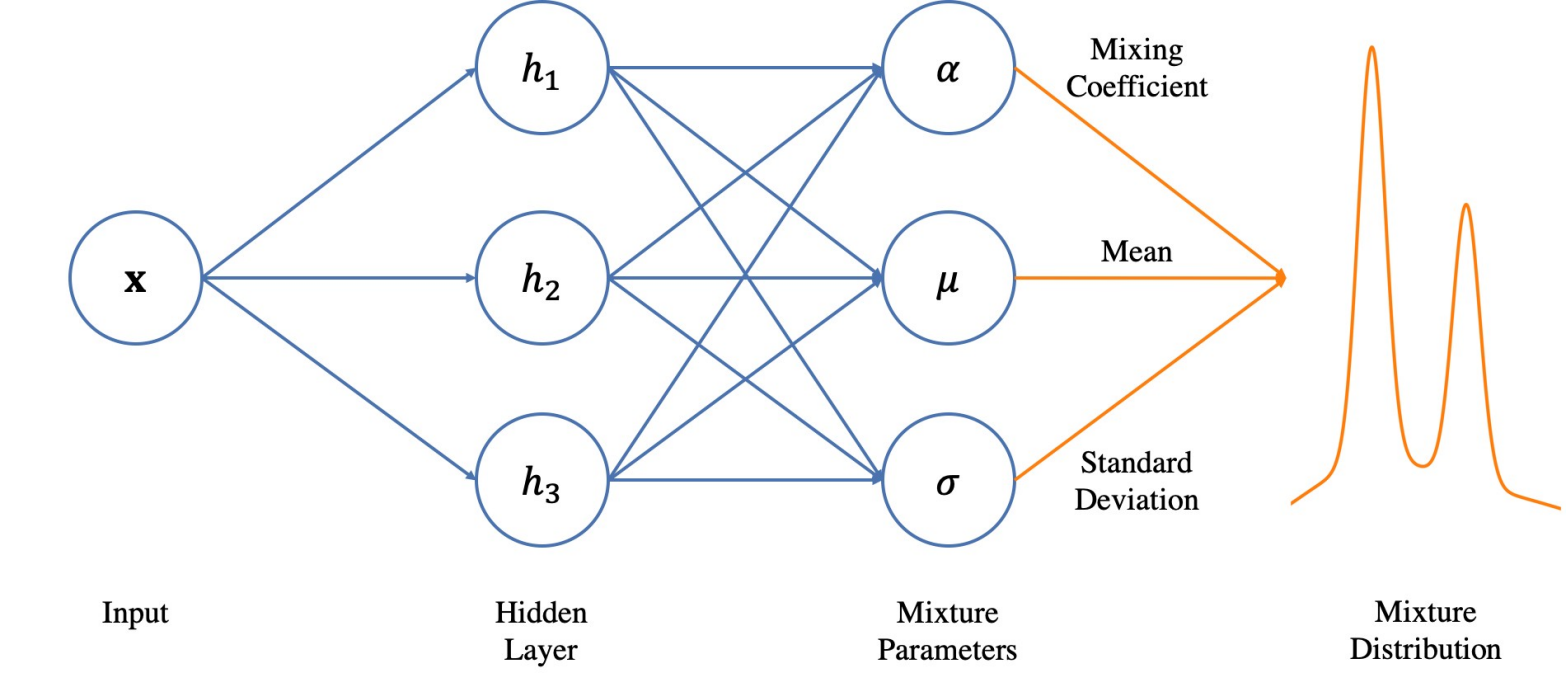
Mixture density networks [38].

MDNs are a combination of a neural network and a mixture of distributions, as represented in Fig 2. In MDNs, neural networks are used to model a mixture of components [37]. The main aspects of MDNs include the type of neural network, the number and size of the hidden layers, the dimension of the output, the number of input parameters, the type of distribution, and the number of distributions [37]. Unlike the LSTM deterministic model with fully determined outputs, MDNs estimate probability distributions of potential outcomes.

In the following equation, the mixture of the probability density function (PDF) p(x) is represented as a combination of the m PDFs with weights Ω = {ω_0_,…, ω_m−1_}, where the sum of weights is equal to 1:

$$p(x)=\sum_{j=0}^{m-1}\omega_{j}p_{j}(x)$$ (11)

Each p_j_ is a normal distribution defined by a variance σ_j_ and a mean μ_j_, according to the following equation:

$$p(x)=\sum_{j=0}^{m-1}\frac{\omega_{j}}{\sqrt{2\pi \sigma_{j}^{2}}}exp(\frac{-1}{2\sigma_{j}^{2}}(x-\mu_{j}{)}^{2})$$ (12)

The model can be fit to the following objective loss function:

$$f(x)=-\sum_{i=0}^{n-1}log\sum_{j=0}^{m-1}\omega_{j}p_{j}(x)$$ (13)

In this study, RNNs are used to output the parameters of a mixture model including the mixing coefficient of each Gaussian kernel (the probability of each kernel), and the mean and variance of each Gaussian kernel.

## COVID-19 predictive models

In this section, the deterministic and stochastic sequence-learning models are explained. These models are used to predict the number of confirmed COVID-19 cases and the effective reproduction numbers in all states in the US. We use data from the Centers for Disease and Prevention website, and have developed a dataset of the number of confirmed COVID-19 cases in all states of the US from January 22, 2020, to November 26, 2020, as indicated in Table 1.

**Table 1:** The confirmed case dataset at one time-step.

Date	Alabama	Alaska	Arizona	Arkansas	California	Colorado	Connecticut	Delaware	Florida	…
3/29/2020	110	12	146	34	480	246	469	18	891	…
…										

Next, we use the EpiEstim package to compute effective reproduction numbers for all time-steps and all states, as represented in Table 2.

**Table 2:** The R_t_ dataset at one time-step.

Date	Alabama	Alaska	Arizona	Arkansas	California	Colorado	Connecticut	Delaware	Florida	…
3/29/2020	2.06	1.89	2.11	1.28	1.77	1.92	2.39	1.91	2.26	…
…										

**Fig 3:**
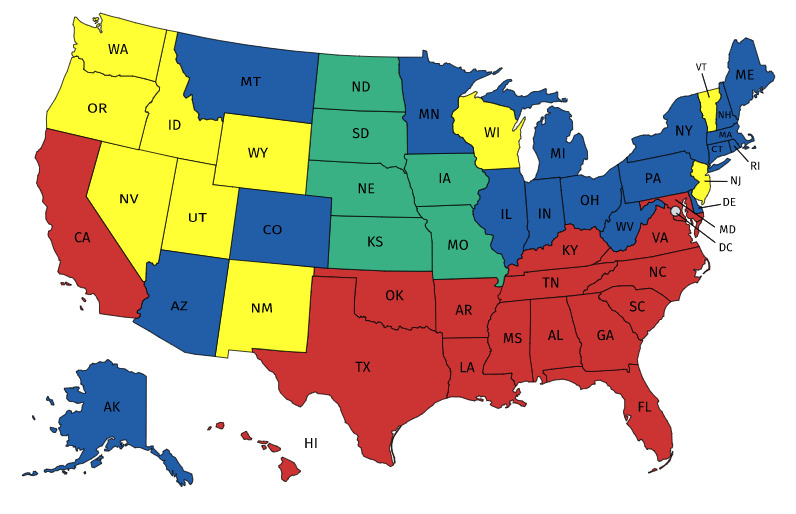
Categorization of all states according to the effective reproduction numbers over time (red: Group one, blue: Group two, green: Group three, yellow: Group four).

Both datasets contain 310 rows (time-step-days) and 50 columns (US states). To decrease the dimensionality of datasets, we use SOM to categorize all states into four categories. We apply the Minisom package [39] to a dataset containing the effective reproduction numbers from August 26, 2020 to November 26, 2020 for all US states. In the dataset, time-steps are considered features, and states are nodes. We have categorized all states into four groups according to the behavior of the effective reproduction numbers over time, as represented in Fig 3.

As shown in Fig 3, most neighboring states are interestingly clustered into the same group, thus indicating that the COVID-19 behavior is similar in close states. This conclusion appears logical, because there is more commuting and traveling between neighboring states.

**Fig 4:**
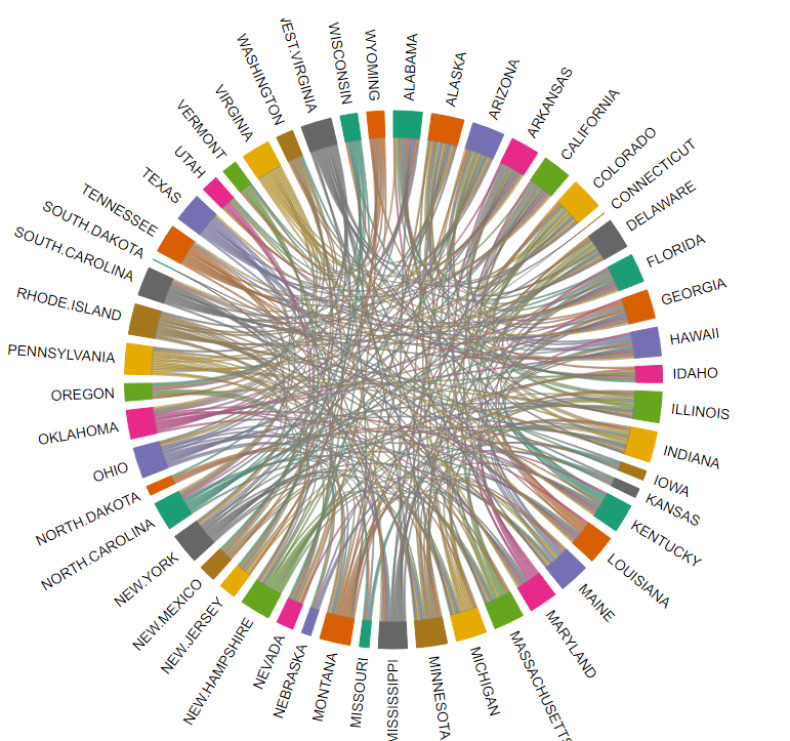
The relationships among states in terms of the similarity of effective reproduction numbers.

We also use the R package Chorddig [40] to visualize all relationships among states according to their similarities in effective reproduction number (Fig 4).

**Fig 5:**
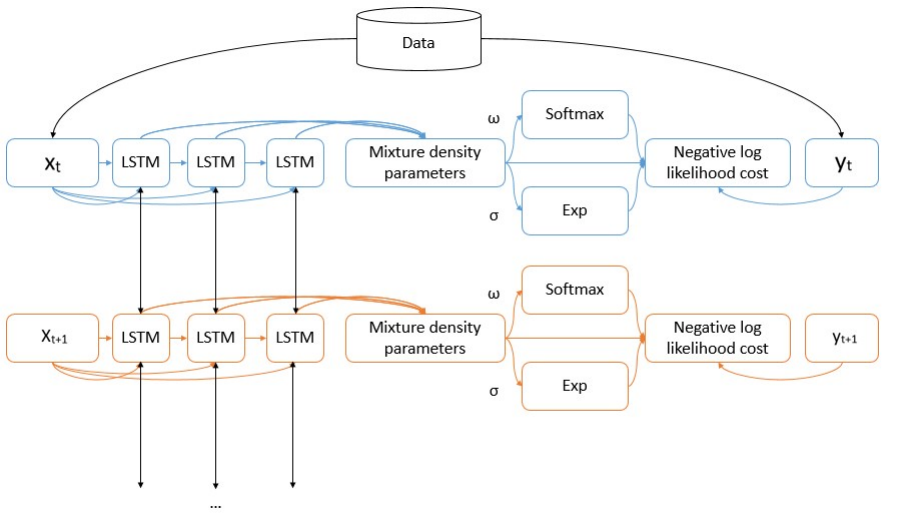
The LSTM-MDN learning model through time-steps.

After categorizing the states into four groups, we select the state with the earliest outbreaks as the leading state in each group. These leading states are used for training the models. Two sequence-learning models are considered: a deterministic LSTM model and a stochastic LSTM/MDN model. Fig 5 represents the structure of the stochastic LSTM/MDN model.

In the stochastic LSTM/MDN model, the neurons corresponding to the means μ_k_(x) are passed to the negative log likelihood cost, but neurons corresponding to the variances σ_k_(x) are passed through an exponential function before moving to the negative log likelihood cost. To satisfy the constraint of a sum of weights equal to 1 (Ω = {ω_0_,…, ω_m−1_}), the neuron corresponding to weights passes through the softmax function. Softmax creates probabilities between 0 and 1 from real values that add up to 1:

$${Softmax(z)}_{j}=\frac{e_{j}^{z}}{\sum_{k=1}^{n}e^{zk}}$$ (14)

As described earlier, the probability density of y_t_ can be calculated according to the following equation:

$$p(y_{t}|x)=\sum_{k=1}^{M}\omega_{k}(x)g_{k}(y_{t}|x)$$ (15)

where g_k_(y_t_|x) is represented in the following equation as the k_th_ multivariate Gaussian kernel.

$$g_{k}(y_{t}|x)=\frac{1}{{\left(2\pi \right)}^{N/2}}exp\left\{\frac{||y_{t}-\mu_{k}^{2}(x)|{|}^{2}}{2\sigma_{k}{\left(x\right)}^{2}}\right\}$$ (16)

where the vector μ_k_(x) is the center of k_th_ kernel. Finally, the error function is represented as follows:

$$E_{t}=-ln\left\{\sum_{k=1}^{M}\omega_{k}(x)g_{k}(y_{t}|x)\right\}$$ (17)

Both deterministic and stochastic models were trained to provide predictions for time-step t + 1 after input of values up to time-step t. However, the output of the LSTM model is a value, whereas the output of the LSTM/MDN model is a mixture density parameters of a Gaussian mixture distribution. Therefore, for the stochastic model, a sample selected from this Gaussian mixture distribution is considered a prediction of the next time-step.

## Experimental study

In this section, the developed stochastic and deterministic models are evaluated on two datasets of confirmed COVID-19 cases and effective reproduction numbers (Please refer to https://github.com/RezaDavahli for models and input data; 10 February 2021). Then they are compared with a linear regression model to better understand their predictive ability. In the next experiment, after performing an Augmented Dickey Fuller test, we remove the seasonality and trend of the non-stationary dataset. We then investigate the performance of the developed models trained on the residuals dataset.

### Experimental setup

The performance of the developed deterministic and stochastic models is evaluated with the datasets of confirmed COVID-19 cases and effective reproduction numbers. The datasets contain values from January 22, 2020 through November 26, 2020 (Please refer to https://github.com/RezaDavahli for models and input data; 10 February 2021). In each dataset, 95% of the data are used for training (including 76% for training and 19% for validation), and 5% are used for testing. The testing set is considered from November 11, 2020 to November 26, 2020. The number of days for the testing set was borrowed from Arora et al. [9] and Hartono [20] aiming to provide comparability of our results. For developing the training dataset, 14 previous days are used in one batch to train the model and predict the value for the next day (1 day in advance). The Tensorflow [41] and Keras [42] libraries are used for developing the networks. The list of parameters in the two models is shown in Table 3.

**Table 3:** List of parameters in the two models.

Elements	LSTM	LSTM/MDN
Time step length	Day	Day
Normalization	Yes	Yes
Number of sequences	14	14
Number of hidden layers	3	2
Number of nodes in each hidden layer	50	10
Number of mixture Gaussian kernels	-	1

### Performance metrics

We use Mean Absolute Percentage Error (MAPE), which is the percentile error of the models, to test the performance of the developed predictive models [43]. As represented in the following equation, y_i, t_ is the real value in state i at time-step t, whereas ŷ_i, t_ is the predicted value.


$$MAPE_{i}=\frac{1}{T}\sum_{t=1}^{T}\frac{\left|y_{i,t}-{ŷ}_{i,t}\right|}{y_{i,t}}$$ (18)

We compare the developed stochastic and deterministic predictions with that of linear regression to better understand the performance of the models.

### Performance results

**Fig 6:**
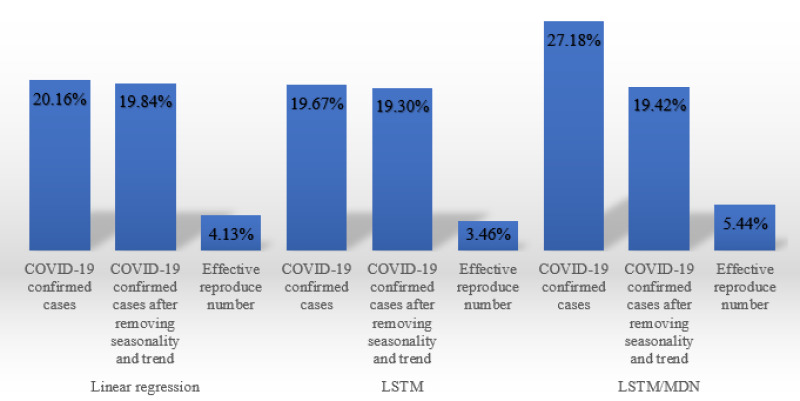
The performance of different combinations of models and datasets.

**Fig 7:**
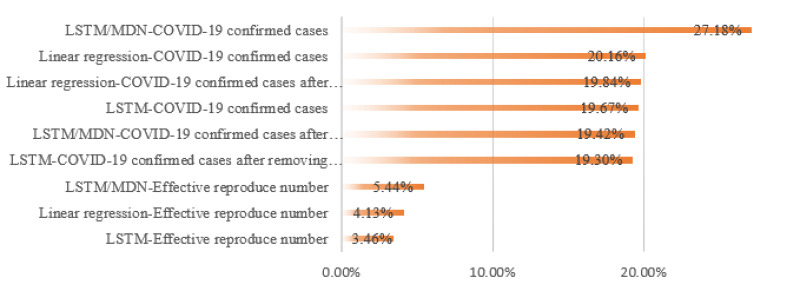
The performance of different combinations of models and datasets.

To fully understand the efficient model, we report the average MAPE for all leading states and for different combinations of models and datasets, as shown in Figs 6 and 7.

Several specific patterns are seen among the data. First, the predictive models trained on effective reproduction numbers showed much better performance than models trained on confirmed cases. On average, there was a 16% difference between the predictions based on confirmed cases versus effective reproduction numbers. Second, unlike the confirmed cases dataset, the R_t_ dataset is stationary, and there is no need to remove the seasonality and trend. However, with the confirmed cases dataset, the greatest improvement in performance due to removal of seasonality and trend was seen in the stochastic LSTM/MDN model. Third, the deterministic LSTM model exhebited the best performance for the two datasets. The LSTM model trained on the effective reproduction number has the best performance, with 3.46% MAPE among all fusions.

**Fig 8:**
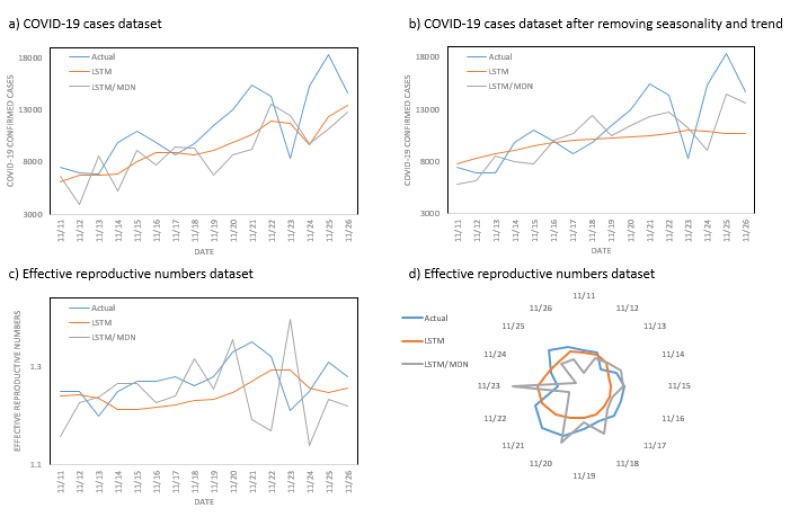
The performance of different combinations of models and datasets in the leading state of California in group one: (a) performance of deterministic and stochastic models trained on the COVID-19 cases dataset, (b) performance of deterministic and stochastic models trained on the dataset of COVID-19 cases after removal of seasonality and trend, (c) performance of deterministic and stochastic models trained on the effective reproduction numbers dataset, (d) performance of deterministic and stochastic models trained on the effective reproduction numbers dataset.

**Fig 9:**
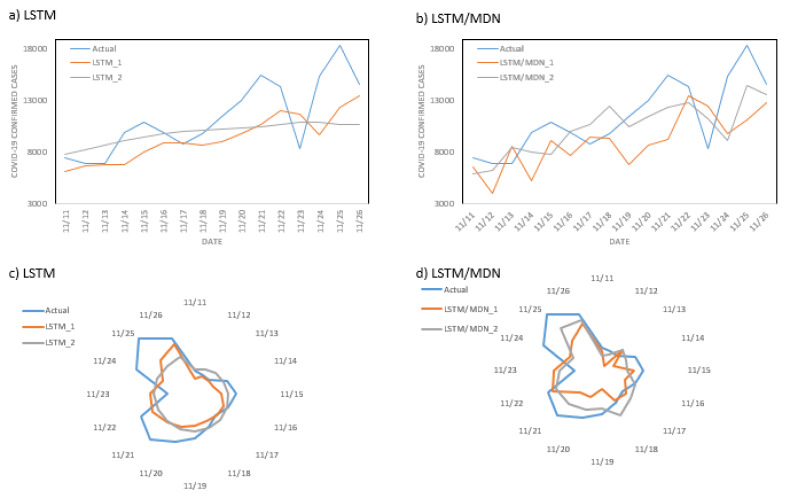
The performance of deterministic and stochastic models trained on the COVID-19 cases dataset with seasonality and trend removed, in comparison to the original dataset in the leading state of California in group one: (a, c) performance of a deterministic model trained on the COVID-19 cases dataset (LSTM_1: without removal of seasonality and trend; LSTM_2: with removal of seasonality and trend), (b, d) performance of the stochastic model trained on the COVID-19 cases dataset (LSTM/MDN_1: without removal of seasonality and trend; LSTM/MDN_2: with removal of seasonality and trend).

We also represented the performance of models from November 11, 2020, to November 26, 2020 in the leading state of California in group one (see in Figs 8 and 9).

As shown in Fig 8, although deterministic LSTM had better performance, stochastic LSTM/MDN was more successful in following the trend of the actual data. However, stochastic LSTM/MDN was much more sensitive to large changes in the actual data. We also show the performance of models on COVID-19 datasets when seasonality and trend are removed in comparison to the original datasets in the leading state of California (Fig 9).

## Limitations

In this study, we developed models to predict the behavior of COVID-19 within the leading US states. Therefore, the main limitation is that we did not consider the effect of states on one another. Many states issued a stay-at-home order, asking residents to stay at home, which reduced mobility between states.

In our subsequent study, we plan to investigate the impacts of mobility on the performance of the sequence learning models.

Although we indicated that the models trained on R_t_ have much better performance, there are some limitations associated with that. The main limitation is that R_t_ can be calculated using different methodologies, which do not give the same estimate. The final major limitation relates to using SOM for dividing US states into four groups. SOM uses an unsupervised learning process to analyze and represent the R_t_ dataset as a map. SOM decreased the dimensionality of the R_t_ dataset by clustering states based on similarities in their respective R_t_ numbers from August 26, 2020 to November 26, 2020. In the resulting map, most neighboring states were clustered together, but there were several exceptions. Because this is an unsupervised clustering technique, the reasoning behind the clusters and exceptions is not clear.

## Conclusion

This study developed stochastic and deterministic sequence learning models based on RNNs and MDNs to predict the behavior of COVID-19 virus in different US states. We trained the models on historical confirmed cases and R_t_ patterns. The developed models can predict geographic spreading of the active virus. The primary dataset contains 310 time-steps and 50 features (US states). To avoid training the models for all states, we used the unsupervised learning methods of SOM to categorize all states into four groups according to their similarity in COVID-19 behavior. After selecting one state from each group as the leading state (the state with the earliest outbreak), we trained the developed models. We found that the predictive models trained on R_t_ have much better performance than those trained on confirmed cases. In addition, the deterministic LSTM model exhibited better performance than the stochastic LSTM/MDN and linear regression models. However, the stochastic model was more successful in predicting the trends in the actual dataset. Finally, LSTM trained on R_t_ showed the best performance, with a MAPE value of 3.46%.
